# Resilience in maternal, newborn, and child health in low- and middle-income countries: findings from a scoping review

**DOI:** 10.1186/s12978-025-01947-w

**Published:** 2025-01-15

**Authors:** Olusesan Ayodeji Makinde, Babasola O. Okusanya, Nchelem K. Ichegbo, Ifeanyi C. Mgbachi, Emmanuel Olamijuwon, Fatima Abdulaziz Sule, Olalekan A. Uthman

**Affiliations:** 1 Department of Research and Development, Viable Helpers Development Organization, PMB 403, Garki Post Office, Abuja, Nigeria; 2 Department of Research and Development, Viable Knowledge Masters, Abuja, Nigeria; 3 Department of Obstetrics and Gynaecology College of Medicine, University of Lagos, Lagos, Nigeria; 4 Department of Global Health, University of Warwick, Coventry, UK; 5 School of Geography and Sustainable Development, University of St Andrews, St Andrews, KY16 9AJ UK

**Keywords:** Maternal health, Newborn health, Child health, Maternal resilience, Child resilience, Scoping review

## Abstract

**Objectives:**

The research objectives were to identify and synthesise prevailing definitions and indices of resilience in maternal, newborn, and child health (MNCH) and propose a harmonised definition of resilience in MNCH research and health programmes in low- and middle-income countries (LMICs).

**Design:**

Scoping review using Arksey and O’Malley’s framework and a Delphi survey for consensus building.

**Participants:**

Mothers, new-borns, and children living in low- and middle-income countries were selected as participants.

**Outcomes:**

Resilience as defined by the authors was deduced from the studies.

**Results:**

Twenty-two out of 76,566 cited studies published between 2006 and 2010 were included in the review. Thirteen (59.1%) examined maternal resilience, and nine (40.9%) examined newborn and child health resilience; most of the included studies were quantitative (n = 17; 81%). Seven studies defined ‘resilience’ in the context of maternal health, most of which described the term at the individual level. ‘Maternal resilience’ was measured using validated scales in five studies; another five defined newborn and child resilience. Only one reviewed study used maternal characteristics to identify newborn and child resilience. The synthesised consensus definition of ‘maternal, newborn, and child resilience’ is* ‘A woman’s ability to prevent or adapt to significant and challenging circumstances including threats, tragedy, and trauma to herself during pregnancy, childbirth, and puerperium and to her neonates or children five years or younger’.*

**Conclusion:**

The information identified was limited but included a few definitions of resilience in MNCH and an index of child resilience in LMICs. The proposed definition is useful for MNCH programme implementation and interventions in LMICs.

*Scoping review registration*: The protocol for this review was registered in the open science framework at the registered address (https://osf.io/jt6nr).

**Supplementary Information:**

The online version contains supplementary material available at 10.1186/s12978-025-01947-w.

## Plain Language Summary

We used a robust search strategy to conduct a scoping review that generated a large amount of literature which we then pruned using a high-performing machine learning algorithm. The study used a novel method to generate and propose a new and uniform definition for resilience in maternal, newborn and child health programmes and research. One important limitation is that this study is a scoping review and did not assess the quality of the studies included in the review. Another limitation is that the definitions were generated from the studies and reports we identified and may have inadvertently missed some publications that are related to our topic because they were not in the databases searched.

**Supplementary Information:**

The online version contains supplementary material available at 10.1186/s12978-025-01947-w.

## Introduction

This century has witnessed increased global attention to the state of maternal, newborn, and child health (MNCH), especially in low- and middle-income countries (LMICs). The United Nations and other developmental agencies committed to the Millennium Development Goals (MDGs) and transitioned their commitments to the global targets of the Sustainable Development Goals (SDGs) in 2016, to be achieved by 2030 [1, 2].

Although maternal indices of the LMICs improved between 2000 and 2020, evidence still indicates weak health systems and poor health indices in many countries. In the world’s least developed countries, the maternal mortality ratio (MMR)—430/100,000 live births—is 30 times higher than that of Europe and Central Asia, which is 13/100,000 live births [3]. While a woman of reproductive age in the least developed countries has a 1-in-56 lifetime risk of dying from a pregnancy-related complication, her counterpart in Australia and New Zealand has a much lower risk of 1:7800 [4]. Sub-Saharan Africa alone has an MMR of 536/100,000 live births and accounts for 66% of all maternal deaths [3]. It is evident that a child’s survival is influenced by the region of the world where they are birthed. Globally, 1.9 million babies are delivered stillborn every year, and 6300 newborn deaths were recorded daily in 2022 [5]. The newborn and under-five mortality rates for that year were 17.3/1000 live births and 37.1/1000 live births, respectively [5]. Globally, 4.9 million under-five deaths (children under five years old) were reported in 2022 [5].

These unpleasant indices speak to a complex mix of challenges in the individual, health system, and policy implementation spheres that need to be addressed for maternal, newborn, and child health SDG targets to be achieved [6]. Although the direct causes of maternal death and causes of newborn and child morbidity and mortality are known [7–9], not every woman or child who experiences death-related health challenges dies from the complications or management of those conditions. Hence, resilience in MNCH is an area to be evaluated, as it might elucidate inroads to attaining the MNCH SDG targets if these factors can be enhanced.

Resilience is generally defined as ‘the process of adapting well in the face of adversity, trauma, tragedy, threats, or significant sources of stress’ [10]. Resilience has been extensively researched in mental health to understand how the interplay of individuals, health systems, and environments leads to mental health conditions [11–13]. The improved understanding of those interactions has led to interventions and programmes that enhance individuals’ coping skills. This knowledge has also reinforced the importance of having health systems to prevent and promptly manage mental health conditions, including leveraging an individual’s resilience factors [14, 15]. In the context of HIV infection, ‘resilience resources’ was defined as ‘positive psychological, behavioural, and/or social adaptations in the face of stressors and adversities’ [16]. The definition of resilience in the context of HIV has been useful in developing interventions that improve compliance to antiretroviral therapy, leading to better health outcomes for people living with HIV infection [16].

However, globally, there is limited research on resilience in the context of MNCH, with sparse research in LMIC settings. As in other contexts, there is no consensus definition or set of indices that identify maternal or child resilience in LMIC populations. This lack of consensus has made planning any interventions for resilience in MNCH difficult. Meanwhile, the COVID-19 pandemic’s impact on fragile maternity and child health services in many LMICs led to adaptations, including the adoption of telemedicine so that services are not entirely truncated [17, 18]. While the use of telemedicine helps fill the immediate gap, it has critical shortcomings for the provision of care in emergencies or critical situations [19]. As of 2019, the number of countries that had not achieved the SDG target of 25 or fewer childhood deaths per 1000 live births was 53 [20], and that number is likely to have risen due to the COVID-19 pandemic. Therefore, concerted efforts, including implementing innovative strategies and additional investments to plug this gap are required to attain the MNCH SDGs. This review research was conceived to guide investments in MNCH interventions and provide a template for improving resilience in mothers, newborns, and children in LMICs. The objectives of this research are to identify and synthesise the prevailing definitions and indices of resilience in MNCH and propose a universal, comprehensive definition of ‘resilience’ for use in MNCH research and health programmes in LMICs.

## Methods

We conducted a systematic scoping review of research on resilience in MNCH in LMICs. Arksey and O’Malley’s framework was used in the research for this study [21]. Building on the framework, we followed five stages in our review: (1) identifying the research question; (2) identifying the relevant studies; (3) selecting the studies; (4) charting data and (5) collating, summarising and reporting results. The search was conducted on 15 January 2021.

We included studies that met the following criteria:

- Population: Pregnant and lactating women, and children under 5 years old, regardless of the child's gender.
- Concept: Studies discussing resilience, or resilient populations in the context of maternal, newborn, and child health (MNCH), including the postpartum period.
- Context: Studies conducted in low- and middle-income countries as classified by the World Bank Country and Lending groups.

We excluded studies that did not focus on MNCH populations, were conducted in high-income countries, or did not discuss resilience.

### Identifying the relevant studies

We developed a detailed search strategy to identify all relevant studies regardless of language or publication status (published, unpublished, in press, or in progress) eligible for this research. The search strategy was structured around three blocks: (1) population (ie, MNCH, health outcomes, healthcare utilisation and social capital), (2) exposure (ie, vulnerability, resilience and high-risk) and (3) setting (ie, low-income and middle-income settings) which also formed our inclusion criteria. Medline, Embase, Scopus, and Web of Science were searched from inception to 7 January 2021 (Appendix 1). We also conducted a wider search that included advanced Google searches for reviews of reports and technical papers from multilateral and bilateral organisations such as USAID, UNICEF, Public Health England, and the World Health Organization (WHO); furthermore, we searched conference proceedings with MNCH themes and reference lists from all the studies and reviews identified using the above methods. The search activities were restricted to low- and middle-income countries, and there were no language restrictions. Appendix 1 shows the full search strategies for Medline and Embase.

### Selecting the studies

A bespoke machine learning algorithm to identify potentially relevant studies was developed to reduce the workload of screening the result from the highly sensitive search [22]. We annotated a random sample of 2500 citations, with only 133 (5%) articles tagged as potentially relevant (‘include’), and 2,367 were labelled irrelevant (‘exclude’; 95%). The bidirectional encoder representations from the transformers (BERT) model was trained and validated as a high-performing algorithm comparable to human screening that attained the desired recall (sensitivity) of at least 95% [23]. The correct prediction to either include or exclude an eligible study at random from the complete database search outputs, based on titles and abstracts, was assessed for machine training and validation of the algorithm. The bidirectional encoder representations from the bespoke BERT classifier demonstrated satisfactory results, with robust F1 scores of 0.97, 0.96, 0.80, 0.96, and 0.96.

Covidence systematic review software was used to manage search outputs and screen eligible studies; for convenience, Airtable® was used for data extraction [24, 25]. Two review authors independently screened the literature search results for potentially relevant studies and obtained the full reports of those studies for further assessment. They independently applied the inclusion criteria to the full-text reports using the eligibility criteria and scrutinised publications to ensure that each study was included in the review only once. Disagreements about eligibility were resolved via consensus within the review team by including a third author in the decision. We also assessed and analysed all excluded studies and the reasons for their exclusion.

### Charting data and analysis

**Fig. 1:**
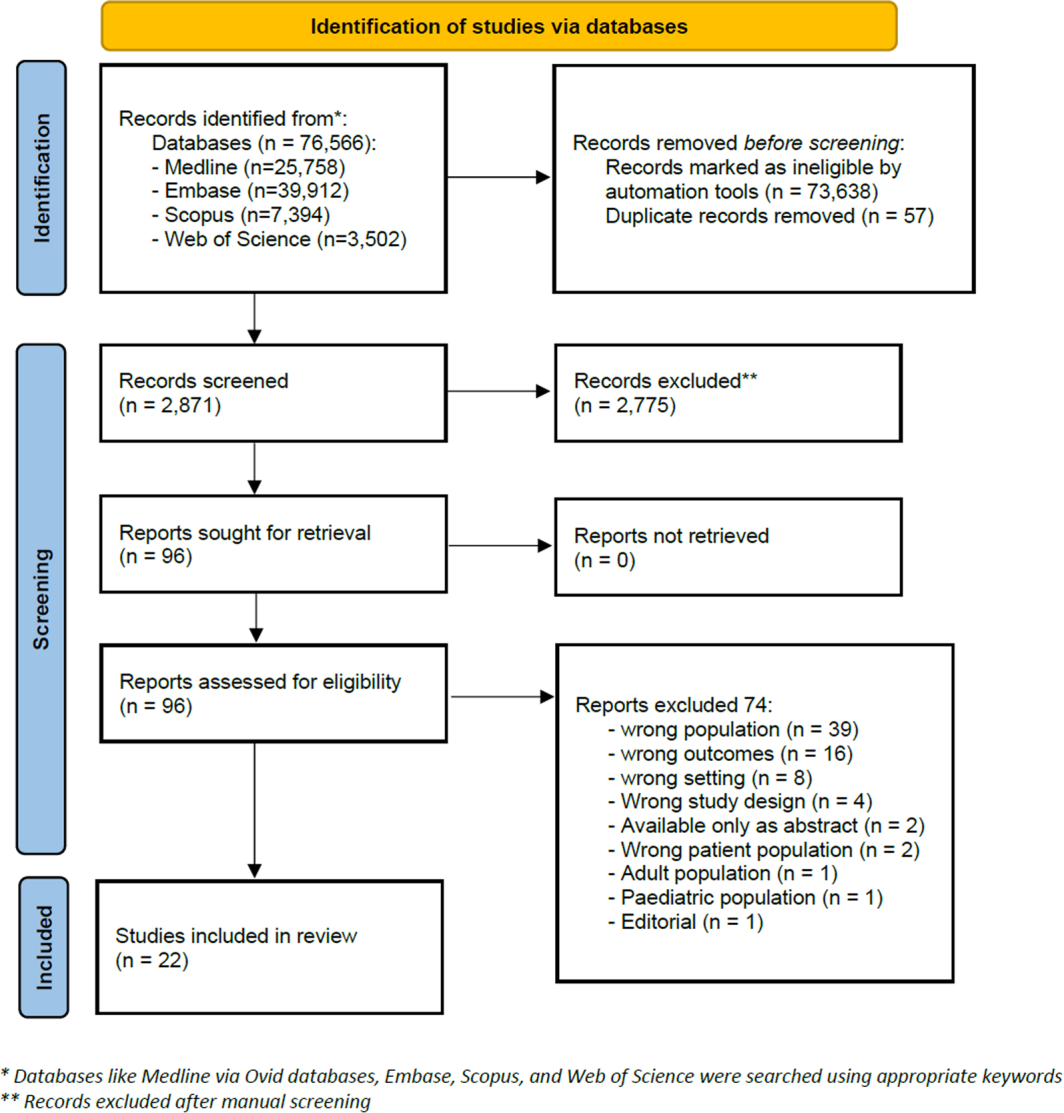
PRISMA study selection process flow chart

Two authors independently charted key information from the included publications. Additionally, two reviewers independently extracted the following data from the included studies: study identification, author(s), publication status, study period, details of the study (study design or type, country, and location of the study), context or setting in which the study was conducted or reported, characteristics of participants, resilience indices, and any definition or validated scale of resilience reported in the study. All disagreements were resolved through discussions between all review authors. Figure 1 illustrates the selection process.

Charted information was collated using Airtable®, downloaded as a Microsoft Excel sheet and used for additional coding and data analysis. Descriptive statistics (frequency and percentage) of country affiliation, publication type, and authors’ institutional affiliations were also calculated. R software was used to generate additional charts and world maps which showed where the studies were conducted.

We extracted and analysed the definitions of resilience used in the included studies. However, the definitions of resilience in MNCH are sparse and heterogeneous. Therefore, key constructs of the identified definitions were used to generate new definitions of resilience in the context of MNCH using word clouds. Definitions generated through these processes were presented to seven experts in MNCH to vote for, in a Delphi survey to help build consensus on the new definitions from the scoping review. The experts were identified from their published papers and invited to participate in the process. We made an effort to ensure an appropriate geographic distribution in the selection of experts. The protocol for this review was registered in the open science framework at the registered address, https://osf.io/jt6nr.

### Patient and public involvement statement

Patients were not involved in the conceptualization or conduct of this study.

## Results

A literature search yielded 76,566 citations. After machine learning and the BERT bespoke classifier were applied and duplicate publications were removed, 2871 titles and abstracts were manually screened. There were 2775 ineligible titles, and 96 articles were selected for full-text screening. Of these, 74 were excluded, and the remaining 22 met the inclusion criteria for the scoping review.

Table 1 presents the characteristics of the included studies. The studies were conducted between 2006 and 2020. There were 13 (59.1%) studies on maternal health resilience and nine (40.9%) on newborn and child health resilience. Most of the studies were quantitative (n = 17; 77%), followed by mixed methods research (n = 3; 13.6%), qualitative (n = 1; 4.5%), and scoping review (n = 1; 4.5%). All the quantitative studies were observational, with 52.9% in the context of maternal resilience (9/17) and 47.1% (8/17) on newborn and child resilience, respectively. Three included studies on maternal resilience and one on newborn and child resilience were published in China [30, 31, 33, 45] all [3] included studies from Brazil examined newborn and child resilience [39, 40, 42]. The countries of publication of other included studies are also shown in Table 1.

**Table 1:** Characteristics of included studies of resilience in maternal, newborn, and child health

S/N	Study ID	Publication country	Research method	Study design	Study objectives
	Maternal Health				
1	Caulker [26]	Sierra Leone	Quantitative	Observational	To compare trends in antenatal care (the first and fourth visit [ANC1 and ANC4]), delivery, and postnatal care (PNC1) service utilisation before, during and after the Ebola outbreak (2014–2016)
2	Gyan^a^ [27]	Ghana	Mixed methods	–	To examine how adolescent girls avoid and/or adjust to the challenges they are predisposed to in their sexual and reproductive experiences
3	Jain [28]	India	Quantitative	Observational	To determine sexual resilience and extrapolate it to others who might not be as successful without an intervention
4	Kishore [29]	India	Quantitative	Observational	To understand the association between life events and depression during pregnancy and if the association is moderated by resilience and social support
5	Li [30]	China	Quantitative	Observational	To examine the relationship between prenatal maternal stress, resilience, and sleep quality; To determine whether resilience plays a mediating role in the relationship between prenatal maternal stress and sleep quality among pregnant women
6	Ma [31]	China	Quantitative	Observational	To explore the effect of resilience to stress on prenatal anxiety/depression in pregnant women
7	Monterrosa-Castro [32]	Colombia	Quantitative	Observational	To evaluate resilience in a group of pregnant adolescents, to estimate the frequency of low resilience level and identify associated psychosocial factors
8	Nie [33]	China	Quantitative	Observational	To examine psychological outcomes influenced by different levels of resilience and explore psychological interactions in threatened preterm labour (TPL) women, spouses, and between women and spouses
9	Nor Jana [34]	Malaysia	Mixed methods	–	To investigate the prediction of mental health by coping, social support, and resilience among unwed young Malaysian pregnant women and mothers
10	Pfeiffer^b^ [35]	Tanzania	Quantitative	Observational	To identify factors that could contribute to strengthening the reproductive resilience of girls in Dar es Salaam, Tanzania
11	Ruzibiza [36]	Rwanda	Qualitative	–	To describe how pregnant teenagers and teen mothers experience stigma in terms of solitude and isolation
12	Subramaney [37]	South Africa	Quantitative	Observational	To gain a better understanding of the circumstances and mental health effects of termination of pregnancy (TOP) in countries where opportunities to terminate pregnancies have only recently become available
13	Warren [38]	Kenya	Mixed methods	Observational	To understand factors that influence repair care-seeking behaviour among women suffering from obstetric fistula in Kenya
	Newborn and child health				
14	Andreani [39]	Brazil	Quantitative	Observational	The specific objective of this study is to build support networks during prematurity for families experiencing preterm births
15	Carvalho-Ramosa [40]	Brazil	Quantitative	Observational	To analyse the microbial community structure changes in breastfed infants of low socio-economic level in early life
16	Chambers [41]	Ukraine	Quantitative	Observational	To examine the consequences of prenatal alcohol exposure that can help identify infants at highest risk and to inform prevention and intervention activities
17	Murray [42]	Brazil	Quantitative	Observational	To identify whether there are sex differences in the association between indices of foetal growth and attention at age four in a population from a middle-income country
18	Theron [43]	SSA	Scoping review	–	To provide evidence on the resilience of children and adolescents living in sub-Saharan Africa to determine what enables their resilience and what may be distinctive about African pathways of child and adolescent resilience
19	Thorne-Lyman [44]	Nepal	Quantitative	Observational	To assess household resilience to risk factors for childhood malnutrition and the change in prevalence of child stunting and wasting in a post hoc sample of areas classified by the government as earthquake areas
20	Wang [45]	China	Quantitative	Observational	To assess the mediated effect of resilience on the relationship of social living ability and child neglect amongst preschool children
21	Worku [46]	Ethiopia	Quantitative	Observational	To investigate the social, emotional behaviour and resilience of orphaned children between 3.5 and 71.8 months of age
22	Yousafzai [47]	Pakistan	Quantitative	Observational	To explore how early nutritional adequacy interventions serve as protective factors and foster the resilience of the child and family to buffer against, ameliorate and recover from nutritional threats and promote healthy development

^a^Includes non-pregnant adolescents with the proportion of pregnant adolescents unreported

^b^16% of participants were pregnant or ever-pregnant young women

### Resilience in maternal health

**Fig. 2:**
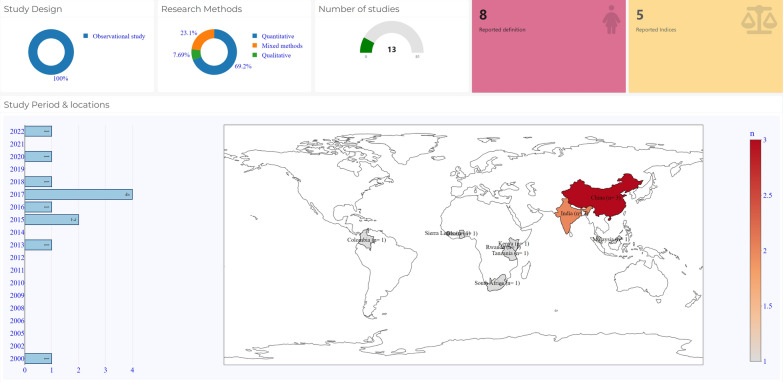
Summary and spatial representation of countries of publication on maternal resilience

Of the 13 publications on resilience in the context of maternal health, six were conducted in sub-Saharan African countries, including Ghana, Kenya, Rwanda, Sierra Leone, South Africa, and Tanzania [26, 27, 35–38]. Two studies were conducted in India, [28, 29] three in China, [30, 31, 33] a study each in Malaysia [34] and Colombia, [32]. See Fig. 2 and Table 1 on the distribution of publications.

The included studies evaluated resilience on the physical and mental health of pregnant women, including pregnant adolescents. Four publications involved pregnant adolescents, young pregnant women or ever-pregnant adolescents. [27, 32, 34, 35]. Jain et al. assessed the sexual resilience of pregnant young women seeking abortion services, [28] while Nie et al. studied the resilience of mothers experiencing threatened preterm labour [33]. Studies on mental health assessed women’s resilience in pregnancy, and stress and sleep quality in pregnancy [30, 31]. Other thematic areas of maternal mental health resilience involved women who just had a termination of pregnancy, [37] depression during pregnancy and service utilisation during the Ebola epidemic [26].

#### Definitions of resilience in the context of maternal health

Seven studies defined resilience in the context of maternal health [29–34, 38]. ‘Resilience is people’s ability to recover, reacquire forces, continue life and project the future despite adversity’ according to Monterrosa-Castro et al. [32] It was also defined as ‘… the capacity that allows an individual to prevent, minimise, or overcome the damage imposed by the adversities of life’ [34]. For survivors of vesicovaginal fistula (VVF), resilience was defined as ‘positive adaptation of those affected by fistula: managing, seeking care, and reintegrating into household and community life’ [38]. For life events and depression in pregnancy, resilience was defined as ‘… entails the process of preventing or attenuating health disturbance after adversity and the process of swift recovery from an adverse condition’ [29]. Resilience was defined as ‘the human ability to adapt in the face of tragedy, trauma, adversity, hardship, and ongoing significant life stressors’ [31] and ‘refers to a prolonged and dynamic process of adapting well in the face of adversity, trauma, tragedy, threats, or even significant sources of stress’ [30].

In addition to the definition of resilience at an individual level, ‘family resilience’ was defined as ‘the characteristics, dimensions, and properties of families, which help families to be resilient to disruption in the face of change and be adaptive in the face of crisis situations’ [33].

#### Indices of resilience in the context of maternal health

None of the included studies used indices to identify maternal resilience. Rather, they used established scales and purpose-built tools to assess maternal resilience.

Five studies used validated scales to measure resilience in maternal health [29–31, 33, 37]. The most commonly used scale was the Connor Davidson Resilience Scale (CD-RISC-10), a 10-item scale [29, 30, 33, 37]. One study used the Resilience Scale for Adults, [31] and two studies used purpose-built tools to assess the resilience of young mothers [27, 35].

Box 1: Indices of the Connor Davidson Resilience Scale [29, 33]

1. I am able to adapt when changes occur.
2. I can deal with whatever comes my way.
3. I try to see the humorous side of things when I am faced with problems.
4. Having to cope with stress can make me stronger.
5. I tend to bounce back after illness, injury, or other hardships.
6. I believe I can achieve my goals, even if there are obstacles.
7. Under pressure, I stay focused and think clearly.
8. I am not easily discouraged by failure.
9. I think of myself as a strong person when dealing with life’s challenges and difficulties.
10. I am able to handle unpleasant or painful feelings like sadness, fear, and anger.

### Resilience in newborn and child health

**Fig. 3:**
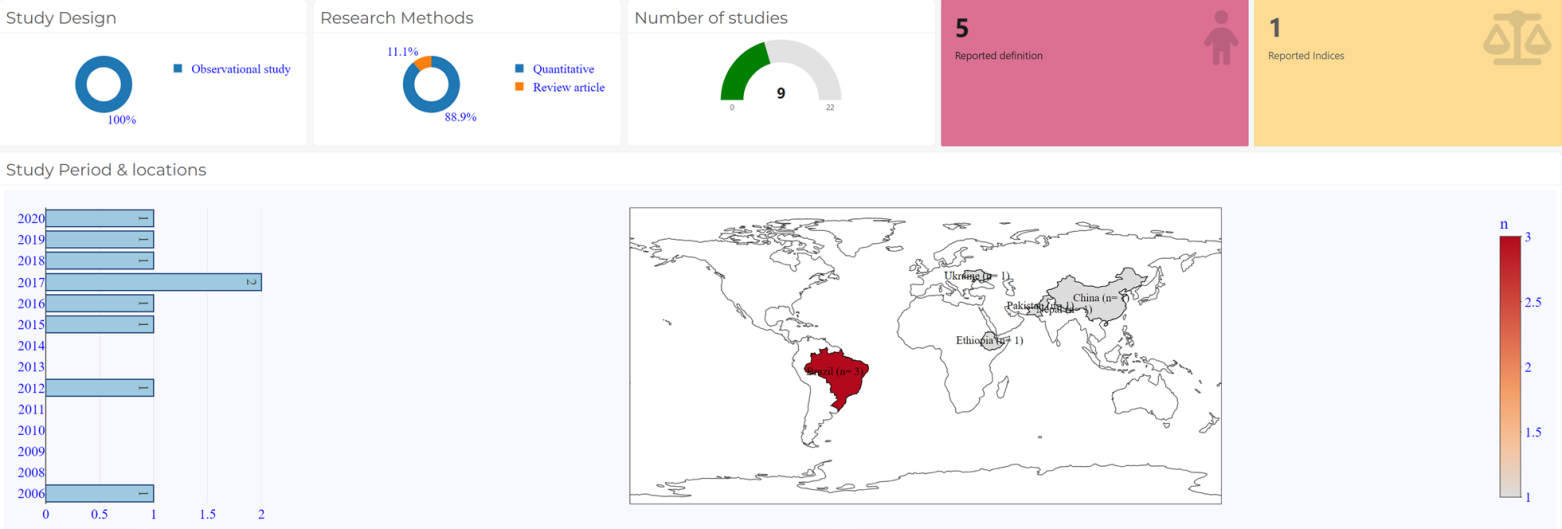
Summary and spatial representation of countries of publication on newborn and child resilience

Nine (40.9%) of the included studies examined newborn and child health resilience, three of which were conducted in Brazil [39, 40, 42] and one study was conducted per each of the following: Ukraine [41], Nepal [44], Pakistan [47], China [47], Ethiopia [46], and South Africa [43]. Figure 3 provides a spatial analysis of the result. Eight of the nine (88.9%) newborn and child health resilience studies included were published in primary research publications [39–47], All of the newborn and child health resilience studies were quantitative and observational in design.

The included studies focused on both physical and mental health resilience. Physical health resilience reports included one regarding building support networks for families experiencing preterm births, [39] one concerning identifying infants at risk of prenatal alcohol exposure, and one regarding the establishment of a prevention/ intervention activity [41]. Other studies focused on microbial resilience to change upon exposure to external factors amongst breastfed infants of women with low socio-economic status, [40] and protective factors that foster childhood and family resilience against nutritional threats [47]. A single study focused on household resilience to childhood malnutrition after an earthquake [44].

The mental health topics examined in some of the studies of childhood resilience included the social and emotional behaviour and resilience of orphans [46] and the mediatory role of resilience on the social living ability of preschool children experiencing neglect [45]. Another study evaluated sex differences in foetal growth and in children at four years of age [42]. Theron’s review article provided evidence regarding resilience in children and adolescents [43].

#### Definitions of resilience in the context of newborn and child health

Five studies provided definitions of resilience in the context of newborn or child health [39, 40, 43, 46, 47]. Resilience was defined as ‘the psychological and biological forces required to successfully go through changes in our lives’ [39]. For breastfed infants from low socio-economic backgrounds, Carvalho-Ramosa defined bacterial resilience as ‘the rate at which microbial composition returns to its original composition after being disturbed’ [40]. Other definitions are ‘the process of adapting and bouncing back from negative emotional experiences, trauma, threats, and adversity’ [46], and ‘the process of, capacity for, or outcome of, successful adaptation despite challenging or threatening circumstances’ [47]. From a socio-ecological perspective, childhood resilience was defined as ‘the process of capacity for, or outcome of, successful adaptation despite challenging or threatening circumstances’ [43].

#### Indices of resilience in the context of newborn and child resilience

One included study used maternal demographic, social, health, and lifestyle characteristics and nutritional status indices to assess newborn and child resilience [41]. Another used the Devereux Center for Resilient Children’s Assessment Tool for Preschoolers for children experiencing neglect [45].

### Proposing new definitions for maternal, newborn and child health resilience

This project was conducted to identify studies on maternal, newborn, and child health (MNCH) resilience that defined resilience along with the constructs of personal agency, family support, and structures. Studies that defined resilience as the personal agency involved both pregnant women and children and supported their views with the need for an adaptation at an individual level to adversities [28–32, 34, 38, 40, 42, 46, 47]. Nie et al. buttressed the importance of family support in MNCH as a source of resilience [33]. Structures and health systems might provide resilience to pregnant women and children [43].

The WHO defines severe maternal outcomes as ‘maternal deaths’ and ‘maternal near misses’. While they define a maternal near-miss as ‘a woman who nearly died but survived a complication that occurred during pregnancy, childbirth, or within 42 days of termination of pregnancy’ [48]. The ICD-11 definition of maternal mortality is:

‘the death of a woman while pregnant or within 42 days of termination of pregnancy, irrespective of the duration and site of the pregnancy, from any cause related to or aggravated by the pregnancy or its management but not from accidental or incidental causes’.[48]

**Fig. 4:**
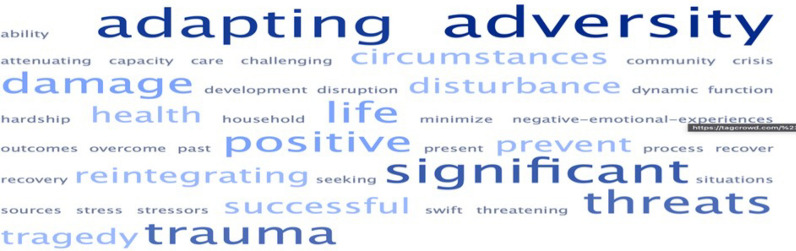
Word cloud of constructs for defining resilience

Synthesizing definitions of resilience in published studies, as was the intention for this project, was difficult because of the different contexts in which resilience in MNCH was assessed. Therefore, the main constructs used in the various definitions were identified and used to generate new definitions of resilience in MNCH. Figure 4 shows the word cloud image which indicates the most common and the lesser used words in available definitions.

The proposed definitions for resilience in maternal, newborn, and child health (MNCH) were as follows:

1. Women’s ability to prevent or adapt to significant and challenging circumstances, including threats, tragedy, and trauma during pregnancy, childbirth, and the puerperium, or in their neonates and children aged five years or younger.
2. The successful, positive adaptation of women to adversity during pregnancy, childbirth, and the puerperium or in their neonates and children aged five years or younger.
3. The dynamic process of recovery of women from significant adversity, including the reintegration into the household and community, leading to successful outcomes during pregnancy, childbirth, and the puerperium, or in their neonates and children aged five years or younger.
4. The life process by which women adapt to or prevent significant threats, tragedy, or trauma leading to successful outcomes during pregnancy, childbirth, and the puerperium, or in their neonates and children aged five years or younger.

The results of the ranking of definitions by MNCH experts engaged in stratifying the definitions, with average ranking scores across them, are presented in Appendix 2. The consensus definition of the process is as follows:

‘‘A w*oman’s ability to prevent or adapt to significant and challenging circumstances including threats, tragedy, and trauma to herself during pregnancy, childbirth, and puerperium and to her neonates or children five years or younger”.*

Women’s ability to prevent or adapt to adversities and processes of recovery is influenced by each woman’s personal (personal agency), relational (partner/husband, and family), and structural (community, environment, and health systems) factors. This diverse array of influences might explain the different outcomes for women with similar adversities.

## Discussion

### Main findings

The objectives of this study were to identify and synthesise definitions and indices of resilience in the context of maternal, newborn, and child health in LMICs and propose a definition that may be used for MNCH programme implementation and interventions. We included 22 publications, with seven studies providing definitions of maternal resilience [29–34, 38], and five providing newborn and child resilience [39, 40, 43, 46, 47]. None of the included studies on maternal resilience used indices to identify resilience in mothers, while one study on newborn and child resilience used maternal characteristics to identify child resilience [41]. Additionally, a new definition of maternal newborn and child resilience was proposed as ‘*A woman’s ability to prevent or adapt to significant and challenging circumstances including threats, tragedy, and trauma to herself during pregnancy, childbirth, and puerperium and to her neonates or children five years or younger.’*

LMICs contribute disproportionately to the global burden of maternal, newborn, and child morbidity and mortality [3, 49]. In these settings, mothers are responsible for caring for themselves, their children, and in some instances, their husbands [50]; thus, the women must adapt and adjust dynamically to life’s stressful events, including pregnancy complications, child illnesses, and natural disasters.

Having a relatively high level of education, being married, and having a source of income might provide some leverage to maternal resilience and enhance a woman’s personal agency [51]. Yet these maternal attributes may be eroded in the face of severe and sudden stressful events, which is when the relational support of her husband, family, community, including the health and financial systems of her community, is critical to ensuring the sustenance of her resilience.

It is important to emphasise that the phrase ‘a woman’s ability to prevent or adapt to significant and challenging circumstances’ in the new MNCH-context definition of ‘resilience’ does not place a responsibility on mothers to be resilient. Rather, her ability to prevent or adapt to stressful life events is intrinsically linked to her support systems, including a good health system and a good financial environment; these resilience mediators are interdependent such that one is able to accommodate or make up for the weakness of another. For instance, a well-educated woman with financial autonomy from her husband may decide to use a private health facility to avoid the challenges inherent in using public health services in LMICs. However, in these settings, most women use public health services, although private healthcare service share for MNCH has been noted to be rising [52]. Therefore, even for women with low personal agency, the relational structures and community structures, as well as the health system structures, should be sufficient for negating the adverse effects on their personal agency, supporting them and their children to create good health outcomes.

It must be stressed that the concept of resilience, including in the MNCH context, is not straightforward, as there is ambiguity in the phrasing ‘to establish resilience' [53]. Masten described resilience as ‘a dynamic system's capacity to withstand or recover from significant threats to its stability, viability, or growth’, and as ‘the ability, processes, or outcomes of effective adaptation in the face of significant threats to function or growth’ [54]. Also, Lutha and Cicchetti described resilience as a ‘dynamic mechanism by which individuals demonstrate positive adaptation in the face of severe adversity or trauma’ [55]. Their explanation of resilience implies that reliance in maintaining health is dependent upon both the individual’s inherent ability and the environment. Similarly, in the context of HIV, resilience of people living with HIV infection has been proposed to align with the socio-ecological model of health to harness interpersonal, neighbourhood/community, and health system resources to attain good health outcomes for them [16]. Also, personal agency (personal negotiation skills/appropriation of resources), relations (supportive adults), and structures (health services) have been reported as building blocks for resilience to adolescents [56]. Our new proposed definition for maternal, newborn and child resilience encompasses personal, relational, community, and health system mediators and buttresses the emerging consensus that resilience mediators should be more than at the individual level.

In one study, women exposed to adversity demonstrated an ability to adapt and become less vulnerable, eventually becoming resilient [57]. This is an important characteristic that, if enhanced, can help vulnerable women increase their level of tolerance and reduce the risk of morbidity or death from such adverse situations. For instance, bonding between a mother and her infant has been shown to increase resistance to the risk of depressive disorder when in adverse life circumstances [58]. While some researchers portray resilience as a self-sufficient entity without the influence of relationships, a feminist viewpoint of resilience is that it is relational and ongoing, not merely a return to equilibrium [59]. This is because of the result of a dynamic relationship between inner strengths and external assistance over the course of a person's life [59]. Coutu et al. asserts that resilient people exhibit three characteristics: acceptance of reality, a solid belief system founded on firmly held values, and the capacity to improvise or adapt. [60].

Children are considered resilient if they overcome severe hardship and stress without obvious physical manifestations [61]. Children's resilience is a dynamic operation, not a fixed trait. As a result, resilience can be created [61, 62]. Building resilience entails minimising risks and increasing protective factors in a child's environment. Since the 1970s, an extensive body of literature has focused on children's resilience in the face of severe adversity. Children's resilience is due to the existence of some attributes and protective factors [63]. The most prominent coping mechanism shared by resilient children comes from one supportive and dedicated adult, whether a parent, caregiver, or another adult [62]. Efforts to foster resilience should be directed toward areas where they can be most effective [55].

Resilience can be enhanced in several ways. Using obstetric haemorrhage, the leading cause of maternal mortality as a case study [64], women within the reproductive age group who might get pregnant should start a pregnancy with normal blood levels. To achieve this, nutritional interventions for women of reproductive age and other pre-conception care should be emphasised in LMIC settings. Providing information on the advantages of pre-conception care, the need for women wanting pregnancy to use pre-conception care services, during which they will be certified to be in good health for pregnancy, should be advocated. Those found with suboptimal health for pregnancy should subsequently be optimised by addressing any identified risks before conception. Pregnant women should have good dietary intake, register for antenatal care, preferably in the first trimester of pregnancy, and consistently use prescribed blood-forming medications and drugs for the intermittent preventive therapy for malaria in pregnancy [65]. Furthermore, health facilities should have personnel knowledgeable about evidence-based interventions to prevent anaemia in pregnancy, skills to manage obstetric haemorrhaging, and life-saving consumables, including a well-stocked blood bank to manage obstetric haemorrhage. This case study shows how a combination of resilience mediators will influence the prevention of—or recovery from—a significant situation to attain good health outcomes, including reduced risk for mortality.

As the appreciation of vulnerability in regard to women and children is required to understand the need for efforts to support resilience in MNCH, vulnerability in MNCH has been extensively discussed as part of this overall research project [66]. In addition, an analysis of the gender dimensions of vulnerability and resilience has been investigated [50]. A framework of sociodemographic indices of vulnerability and resilience, with emphasis on mediators of resilience, has also been proposed as part of the project [67], to engender improved maternal newborn and child health outcomes.

While the scoping review focused on resilience in MNCH in LMICs, it is essential to recognize that the findings have significant applicability to high-income countries (HICs) as well. The concept of resilience in MNCH is relevant across diverse economic contexts, as the factors influencing resilience, such as personal agency, family support, and structural factors, are universal [68]. These factors contribute to an individual's ability to cope with and adapt to adversity, regardless of the economic status of their country [69].

However, it is crucial to recognize that the unique challenges faced by women and children in LMICs, such as limited access to healthcare, resources, and education, may necessitate a more targeted approach to understanding and promoting resilience in these settings [47]. The social, economic, and environmental factors that contribute to vulnerability in LMICs can exacerbate the impact of adversity on maternal and child well-being [70]. Therefore, research focused on resilience in MNCH in LMICs is essential to identify context-specific factors and develop interventions tailored to the needs of these populations.

In HICs, women and children may face unique challenges that can impact their resilience, such as work-life balance issues, social isolation, and mental health concerns [71]. For example, in the United States, the prevalence of postpartum depression ranges from 10 to 20%, [72] which can have significant consequences for maternal and child well-being. Research has shown that maternal resilience can serve as a protective factor against postpartum depression and promote positive child development outcomes [73].

Similarly, a study by Sójta et al. [74] found that lower levels of resilience during pregnancy may be a significant predictor of increased severity of depressive symptoms and higher levels of anxiety related to childbirth among the perinatal population in Poland, highlighting the importance of resilience in promoting maternal mental health in HIC. The study also identified social support as a key factor in fostering resilience, emphasizing the role of family and community in promoting maternal well-being.

In addition to maternal resilience, child resilience is a critical area of research in HICs. Children in HICs may face adversities such as poverty, family instability, and exposure to violence, which can have lasting effects on their development and well-being [75]. A study followed a cohort of children from birth to adulthood and found that one-third of the children who were exposed to significant adversity demonstrated resilience and were able to overcome their challenges [76]. The study identified several protective factors, such as a supportive family environment and strong social connections, that contributed to their resilience.

Research on resilience in MNCH in HICs can provide valuable insights into the universal aspects of resilience and inform the development of interventions and policies aimed at promoting maternal and child well-being. For example, a systematic review of resilience-promoting interventions for children and families in HICs, found that effective interventions targeted multiple levels of influence, including individual, family, and community factors [77]. The study highlighted the importance of a holistic approach to promoting resilience, which is relevant across diverse economic contexts.

Furthermore, comparative studies examining resilience in MNCH in both LMICs and HICs can contribute to a more comprehensive understanding of the factors that influence resilience across diverse settings. A study by Ungar et al. examined resilience among youth in 14 communities across 11 countries, including both LMICs and HICs, and found that resilience was influenced by a complex interplay of individual, family, and community factors [78]. The study highlighted the importance of considering cultural and contextual factors in understanding and promoting resilience.

While this scoping review focused on resilience in MNCH in LMICs, the findings have significant applicability to HICs as well. Research on resilience in MNCH in HICs can provide valuable insights into the universal aspects of resilience and inform the development of interventions and policies aimed at promoting maternal and child well-being. Comparative studies examining resilience in MNCH in both LMICs and HICs can contribute to a more comprehensive understanding of the factors that influence resilience across diverse settings. By recognizing the relevance of resilience in MNCH across economic contexts, researchers and policymakers can work towards developing a global framework for promoting maternal and child well-being. Future research could explore the similarities and differences in resilience factors across diverse economic contexts to further inform interventions and policies aimed at enhancing MNCH resilience globally. Comparative studies examining resilience in MNCH in both LMICs and high-income countries could provide valuable insights into the universal and context-specific aspects of resilience [79]. Such research could contribute to the development of a more comprehensive framework for understanding and promoting resilience in MNCH across diverse settings.

Although the proposed definition of resilience in MNCH emphasizes the woman's ability to prevent or adapt to significant challenges, it is crucial to recognize the interdependence of maternal, newborn, and child resilience. The well-being and resilience of newborns and children are closely tied to the resilience of their mothers or primary caregivers [80]. Maternal resilience can serve as a protective factor for child development, as it influences the quality of caregiving, attachment, and the overall family environment [81].

While the scoping review did not yield sufficient information to develop separate definitions for newborn and child resilience, it is important to acknowledge that resilience is a dynamic process that evolves throughout an individual's lifespan [82]. The factors contributing to resilience may vary at different stages of child development, and the role of maternal resilience in shaping child outcomes may also change over time [83]. For example, during infancy and early childhood, the mother's ability to provide responsive caregiving and a nurturing environment may be critical for promoting resilience [84]. As children grow older, their own individual characteristics, such as temperament, cognitive abilities, and social skills, may become increasingly important in fostering resilience [85].

Future research should explore the unique factors contributing to resilience at different stages of child development and the role of maternal resilience in shaping child outcomes. Longitudinal studies examining the trajectories of maternal and child resilience over time could provide valuable insights into the dynamic nature of resilience and the interplay between maternal and child factors [86]. Additionally, research investigating the mechanisms through which maternal resilience influences child resilience, such as parenting practices, mother–child interactions, and the transmission of coping strategies, could inform the development of interventions aimed at promoting resilience in both mothers and their children [81].

A more comprehensive understanding of the interplay between maternal, newborn, and child resilience can inform the development of targeted interventions to promote the well-being of both mothers and their children. Interventions that focus on strengthening maternal resilience, such as parenting support programs, stress management techniques, and access to social support networks, may have cascading positive effects on child resilience [87]. Similarly, interventions that target child resilience, such as early childhood education programs and social-emotional learning initiatives, may also indirectly support maternal resilience by reducing parenting stress and enhancing family well-being [80].

One of the objectives of this scoping review was to identify indices of resilience in MNCH in LMICs. However, the review revealed a paucity of studies using standardized indices to measure resilience in this context. The lack of validated resilience indices specific to MNCH in LMICs highlights a significant gap in the literature and presents an opportunity for future research [88]. The development and validation of context-specific resilience indices can help researchers and practitioners assess resilience levels, identify factors contributing to resilience, and evaluate the effectiveness of interventions aimed at promoting resilience in MNCH [89].

The complex nature of resilience, which involves the interplay of individual, family, and community factors, poses challenges for its measurement [90]. Existing resilience indices, such as the Connor-Davidson Resilience Scale (CD-RISC) and the Resilience Scale for Adults (RSA), have been developed and validated primarily in high-income countries and may not fully capture the unique cultural and contextual factors influencing resilience in LMICs [88]. The adaptation and validation of these indices in LMIC settings, as well as the development of new indices tailored to the specific challenges faced by women and children in these contexts, are necessary steps to advance research on resilience in MNCH.

Furthermore, the use of standardized indices can facilitate comparisons across studies and populations, contributing to a more comprehensive understanding of resilience in MNCH globally. The ability to compare resilience levels and identify common resilience factors across different LMIC settings can inform the development of evidence-based interventions and policies aimed at promoting resilience in MNCH [90]. Standardized indices can also enable researchers to track changes in resilience over time, assess the impact of adverse events on resilience, and evaluate the effectiveness of resilience-promoting interventions [89].

In addition to the development and validation of resilience indices, there is a need for more qualitative research exploring the lived experiences of women and children in LMICs and the factors that contribute to their resilience. Qualitative studies can provide valuable insights into the cultural, social, and contextual factors that shape resilience in MNCH and inform the development of culturally sensitive and context-specific resilience indices [90]. A mixed-methods approach, combining qualitative and quantitative methods, can provide a more comprehensive understanding of resilience in MNCH and strengthen the validity and reliability of resilience indices [91].

The paucity of studies using standardized indices to measure resilience in MNCH in LMICs underscores the need for further research in this area. The development, validation, and use of context-specific resilience indices, along with qualitative and mixed-methods research, can contribute to a more nuanced understanding of resilience in MNCH and inform the development of effective interventions and policies to promote the well-being of women and children in LMICs.

### Limitations and strengths

Included studies defined resilience differently, with different connotations making the synthesis of a definition of resilience in MNCH difficult. Additionally, few studies have used a definition in their research. The same applies to the use of indices to identify mothers or children who are resilient. In the systematic review of resilience in HIV, only two studies defined resilience, and the majority examined factors of individual resilience. Another limitation is that the scope of the review was restricted to LMICs. Otherwise, the review’s strengths include a detailed search of the literature, including grey literature, which led to the identification of studies that used personal, interpersonal, community, and health system variables to infer resilience mediators. The review also included young pregnant women, a group often excluded from MNCH research.

Another strength of this review is the generation of suggested new definitions, and the one elected preferred (chosen by consensus of a voting panel of experts), despite the heterogeneity of the definitions and focuses of the included studies. The use of the main constructs of definitions made word cloud aggregation possible to generate new definitions. Finally, for programmatic purposes, the proposed definition of ‘newborn and child resilience’ recognises the upper age limit of five years might be extended to nine years for a child, as proposed by the WHO.

## Conclusion

The scoping review identified a few definitions of resilience in MNCH and an index of child resilience in LMICs. Although it presents contemporary evidence of publications on MNCH resilience in LMICs, the available evidence is limited. In addition, the lack of specific identification criteria has prevented many programmes providing health, educational, psychosocial, and other support services in MNCH from recognising those in greatest need, resulting in ineffective and inequitable resource use. This lack of uniformity might explain, in part, why governments and donors have found it difficult to set priorities, allocate resources, and establish effective strategies for improving maternal, newborn, and child well-being. The definition proposed as a result of this scoping review can support more effective MNCH programme implementation and interventions.

## Supplementary Information

Supplementary Material 1.

## Data Availability

All data relevant to the study are included in the article or uploaded as supplementary information.
